# Supplementary material for: The Targeted Degradation of BRAF V600E Reveals the Mechanisms of Resistance to BRAF-Targeted Treatments in Colorectal Cancer Cells
Source: Cancers (Basel). 2023 Dec 12;15(24):5805. doi: 10.3390/cancers15245805 (PMC10741866; doi:10.3390/cancers15245805)
Supplement: Supplementary file 1 [file cancers-15-05805-s001.zip › cancers-2734117-supplementary.pdf]

Complementary Materials

1. Antibodies used:

Antibody	Vendor	Catalog number
BRAF V600E	Thermo Fisher Scientific	MA5-24661
BRAF	Cell Signaling Technologies	14814S
MEK	Cell Signaling Technologies	4694S
pMEK	Cell Signaling Technologies	9154S
Erk	Cell Signaling Technologies	4695S
pErk	Cell Signaling Technologies	4377S
Akt	Cell Signaling Technologies	4691S
Akt pS473	Cell Signaling Technologies	4060S
Actin	Cell Signaling Technologies	3700S
Casp3	Cell Signaling Technologies	14220S
Cleaved Casp3	Cell Signaling Technologies	9664S
Casp9	Cell Signaling Technologies	9508S
Cleaved Casp9	Cell Signaling Technologies	52873S
PARP	Cell Signaling Technologies	9542S

2. Full images of western blots.

Figure 1A

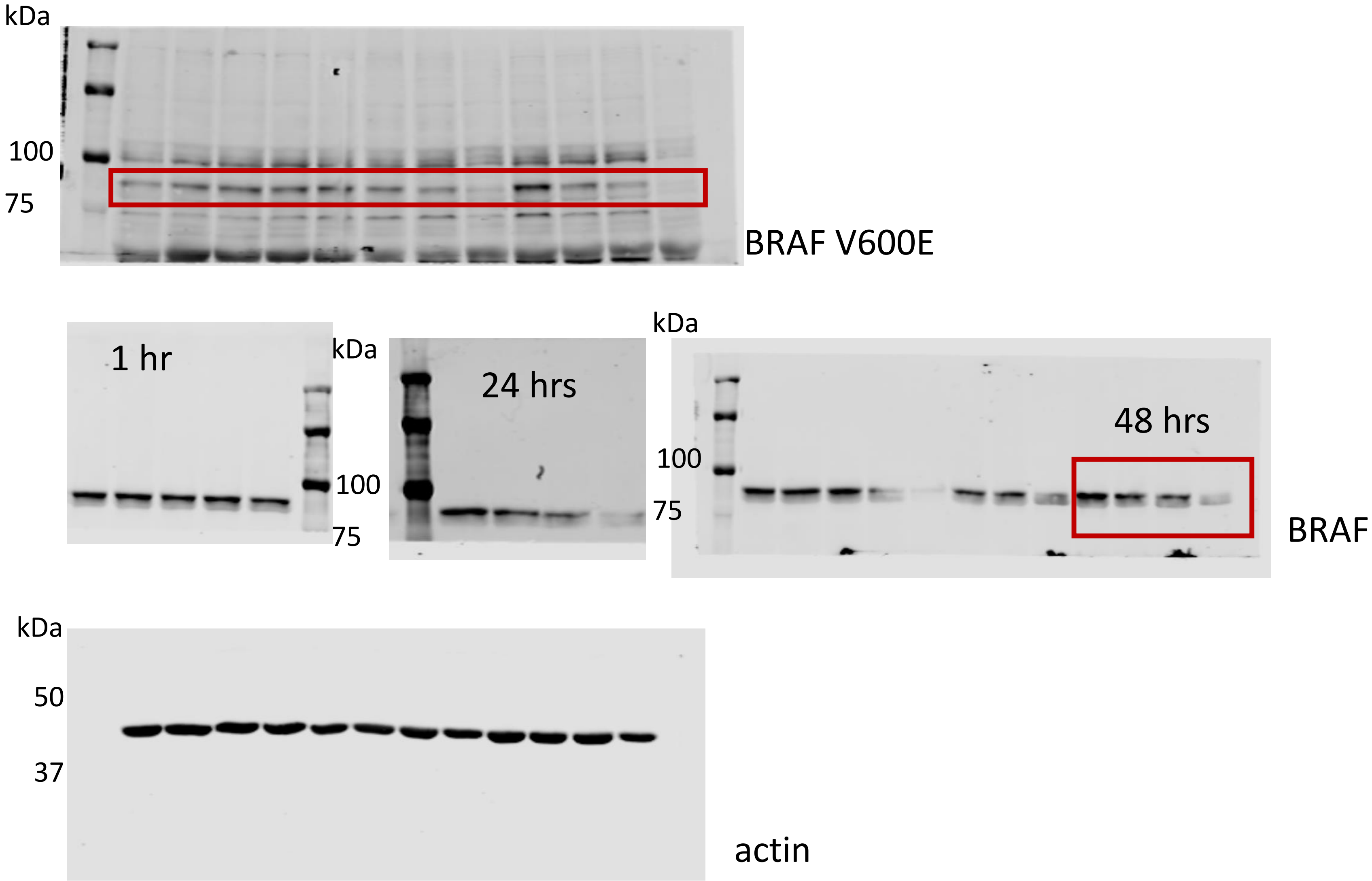

Figure 1B

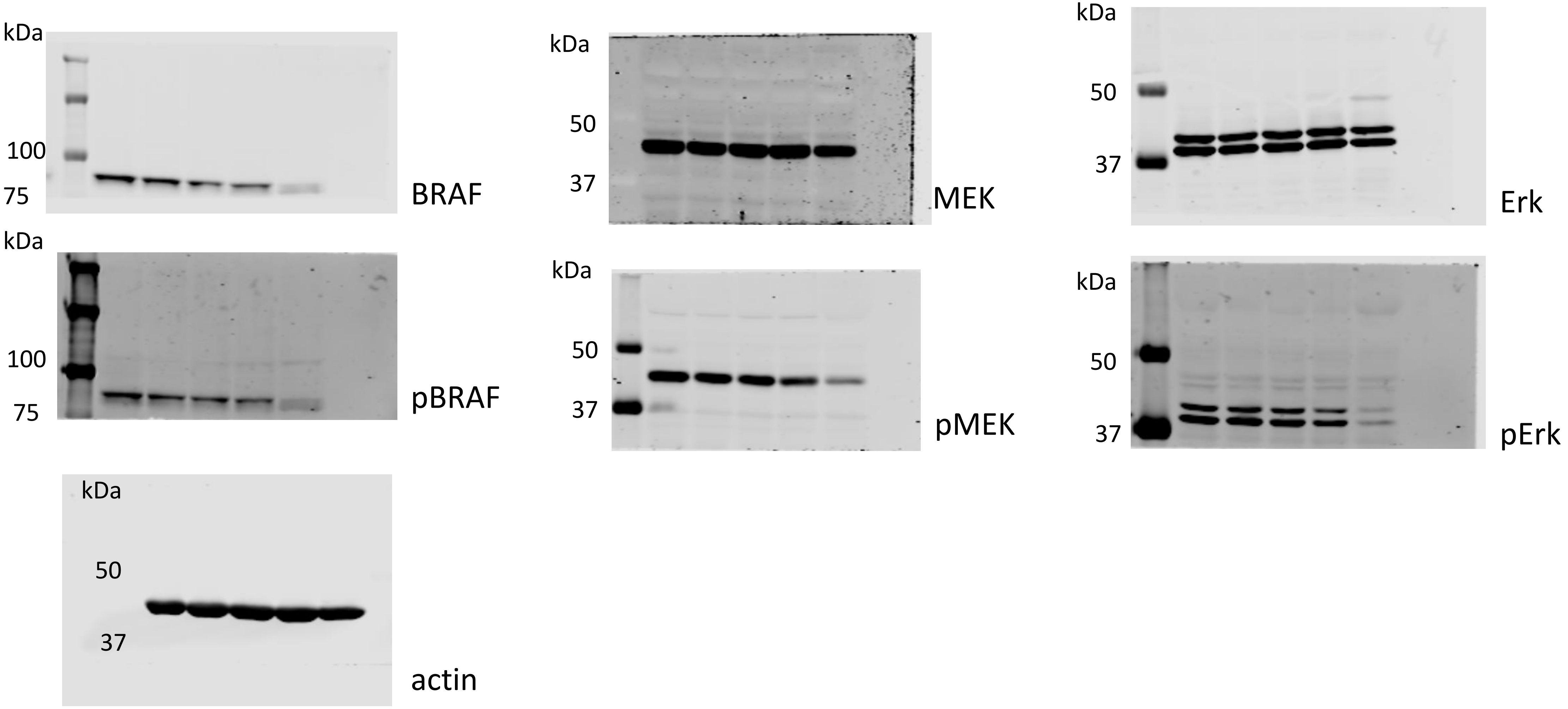
kDa
100
75
BRAF
kDa
100
75
pBRAF
kDa
50
37
actin
kDa
50
37
MEK
kDa
50
37
pMEK
kDa
50
37
Erk
kDa
50
37
pErk

Figure 2-Colo 205

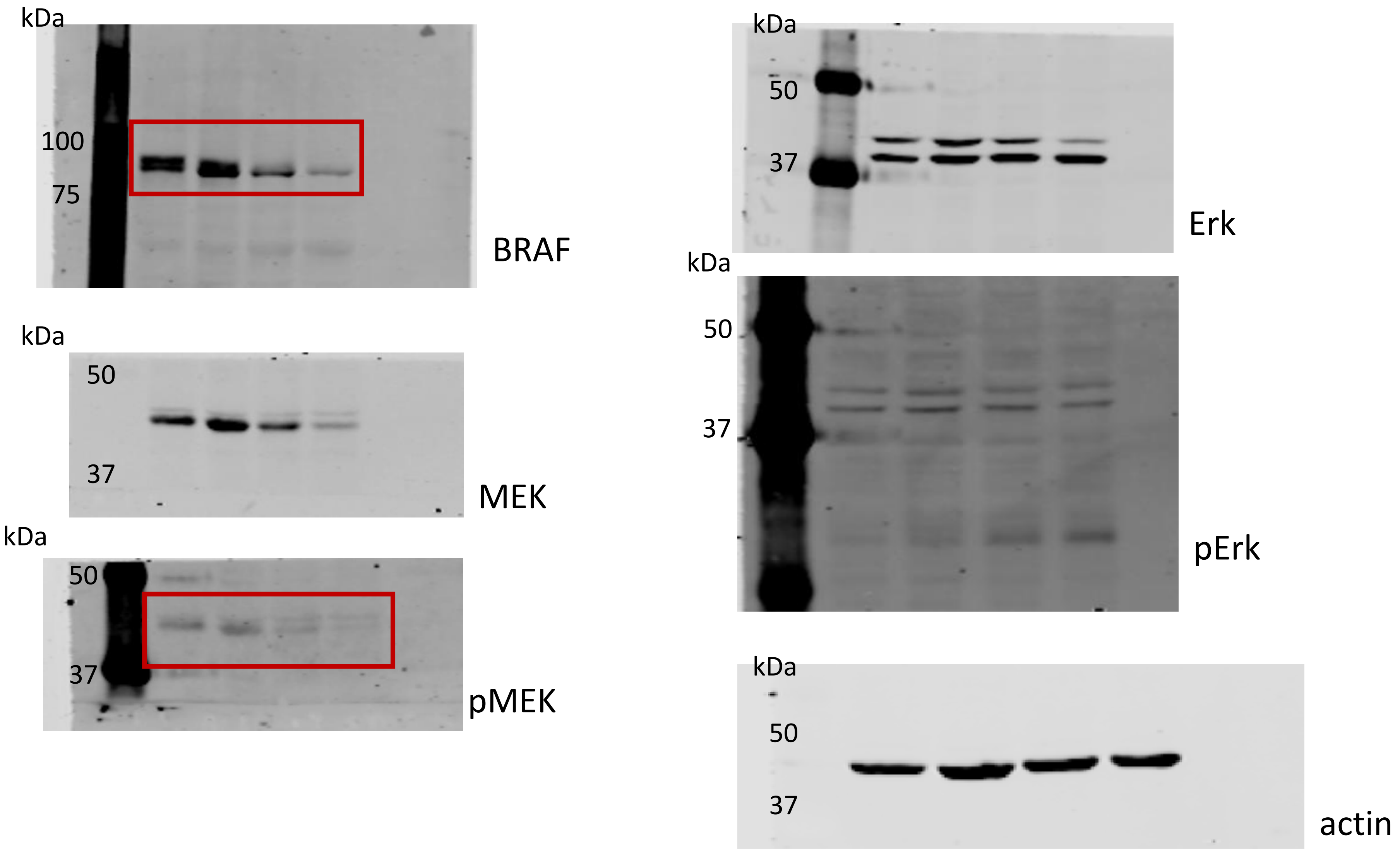

Figure 2-LS411N

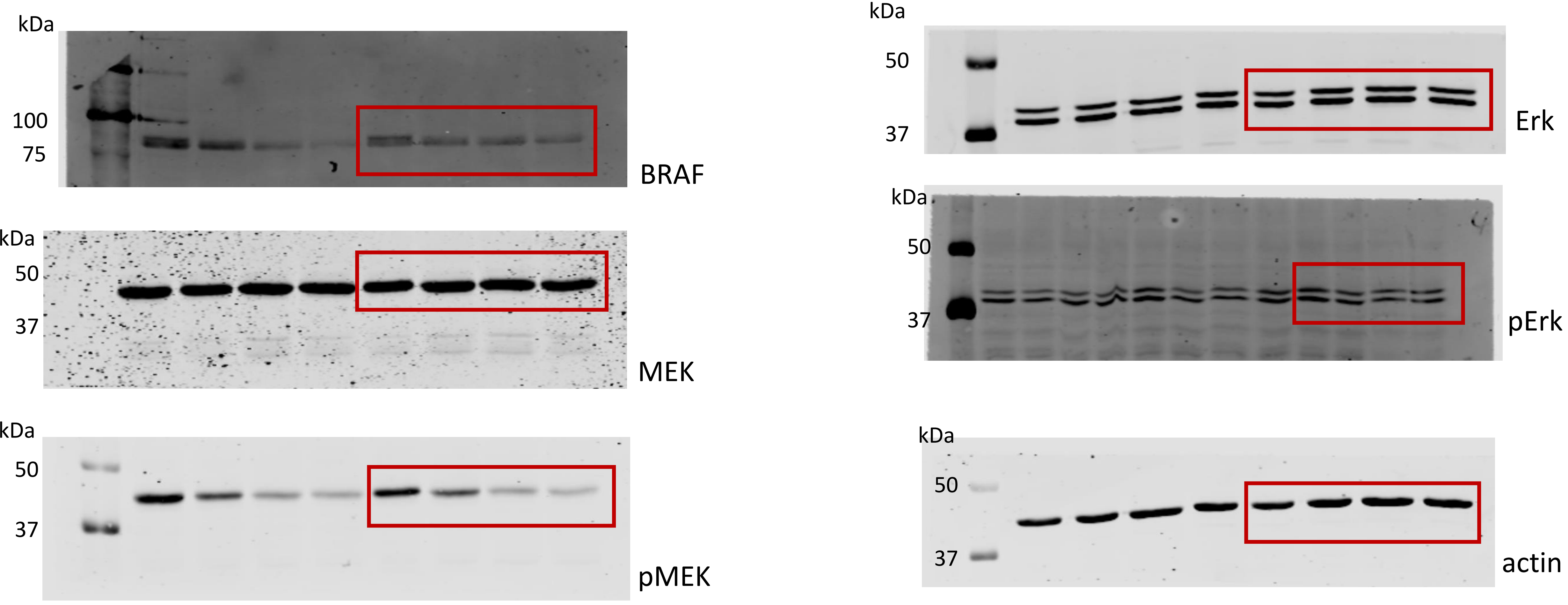

Figure 2-HT-29

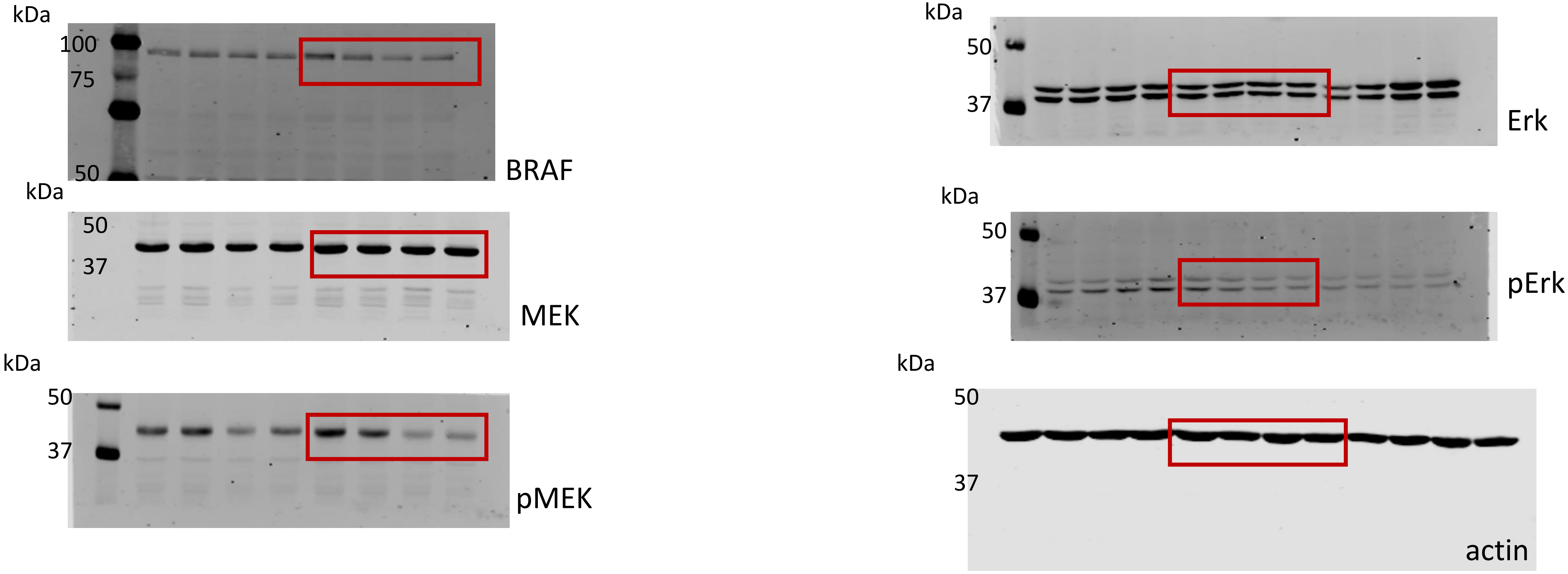

Figure 2-RKO

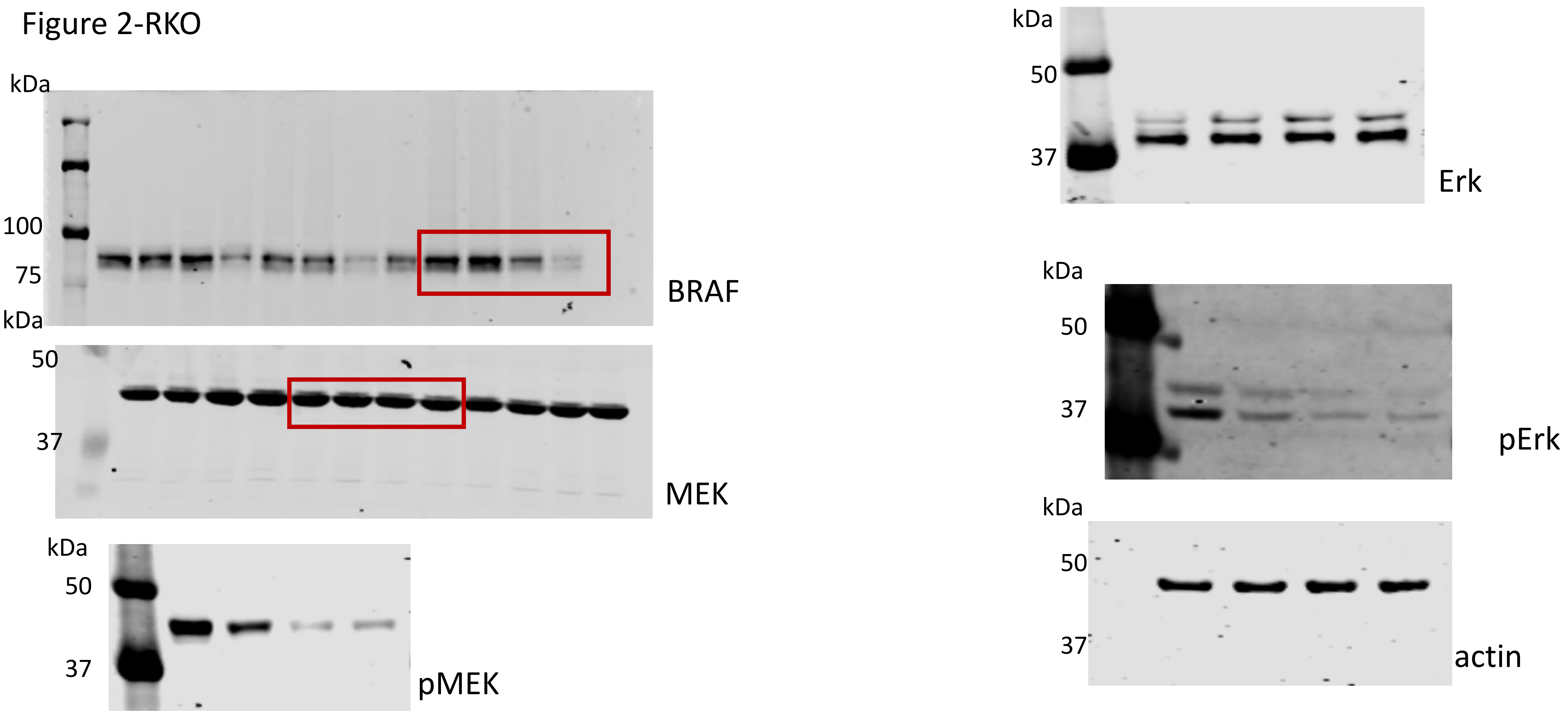
kDa
100
75
BRAF
kDa
50
37
MEK
kDa
50
37
pMEK
kDa
50
37
Erk
kDa
50
37
pErk
kDa
50
37
actin

Figure 4B

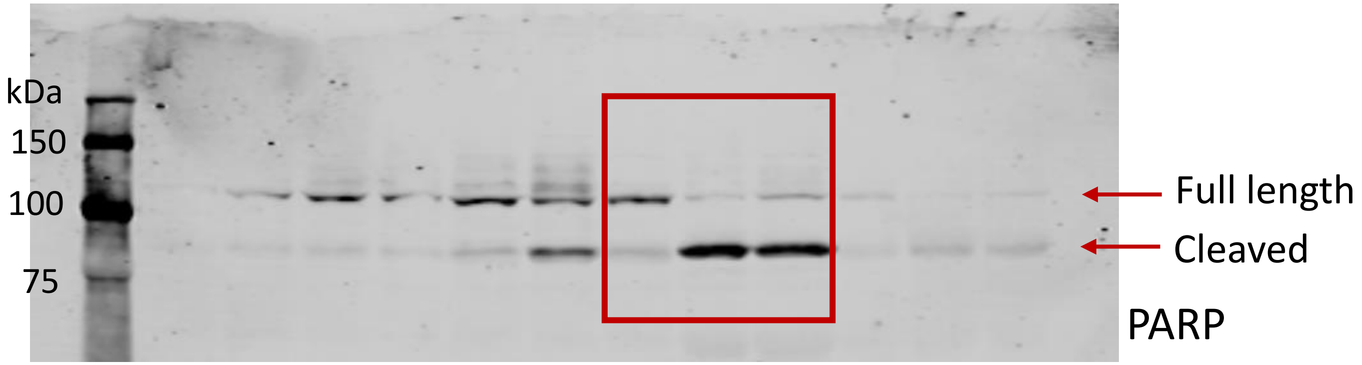
kDa
150
100
75
Full length
Cleaved
PARP

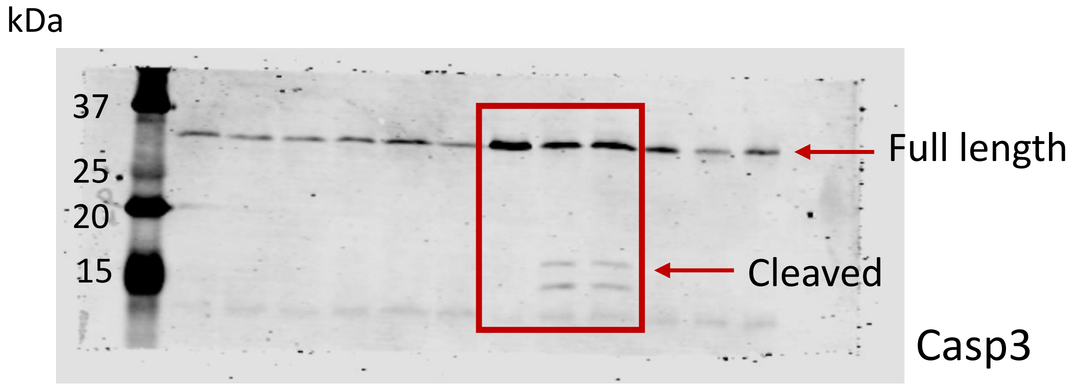
kDa
37
25
20
15
Full length
Cleaved
Casp3

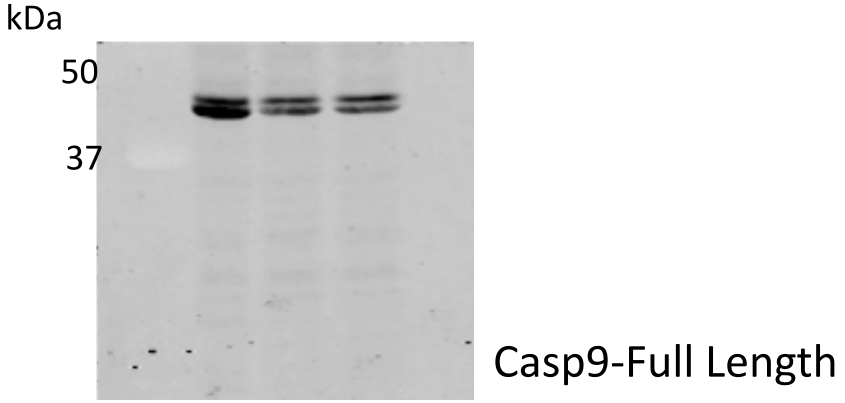
kDa
50
37
Casp9-Full Length

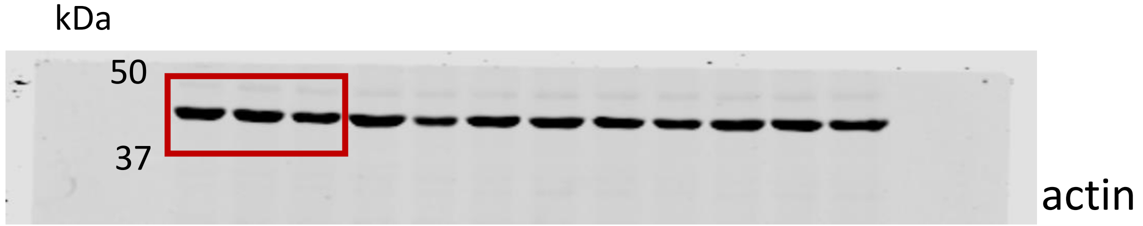
kDa
50
37
actin

Figure 4D

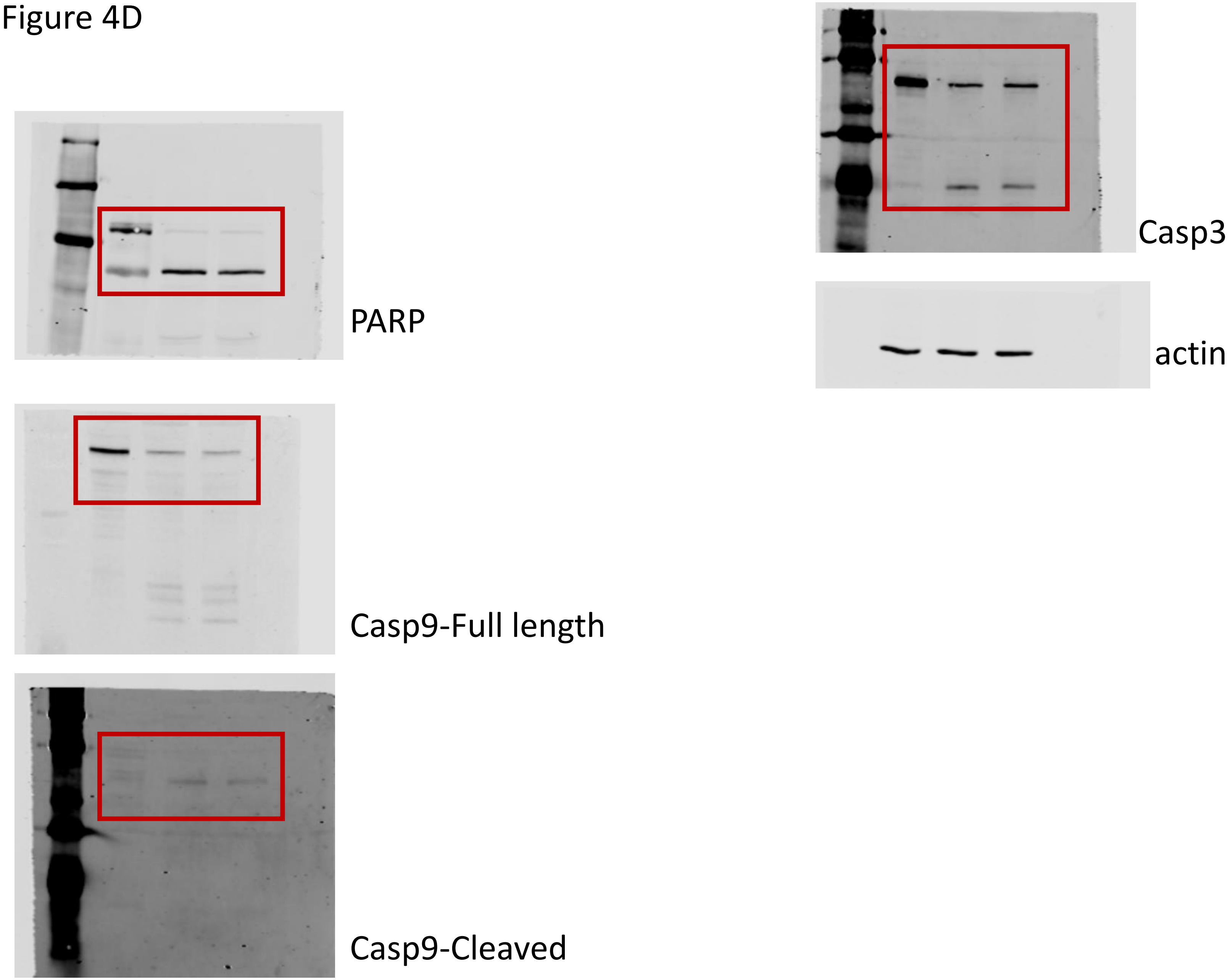

Figure 5D

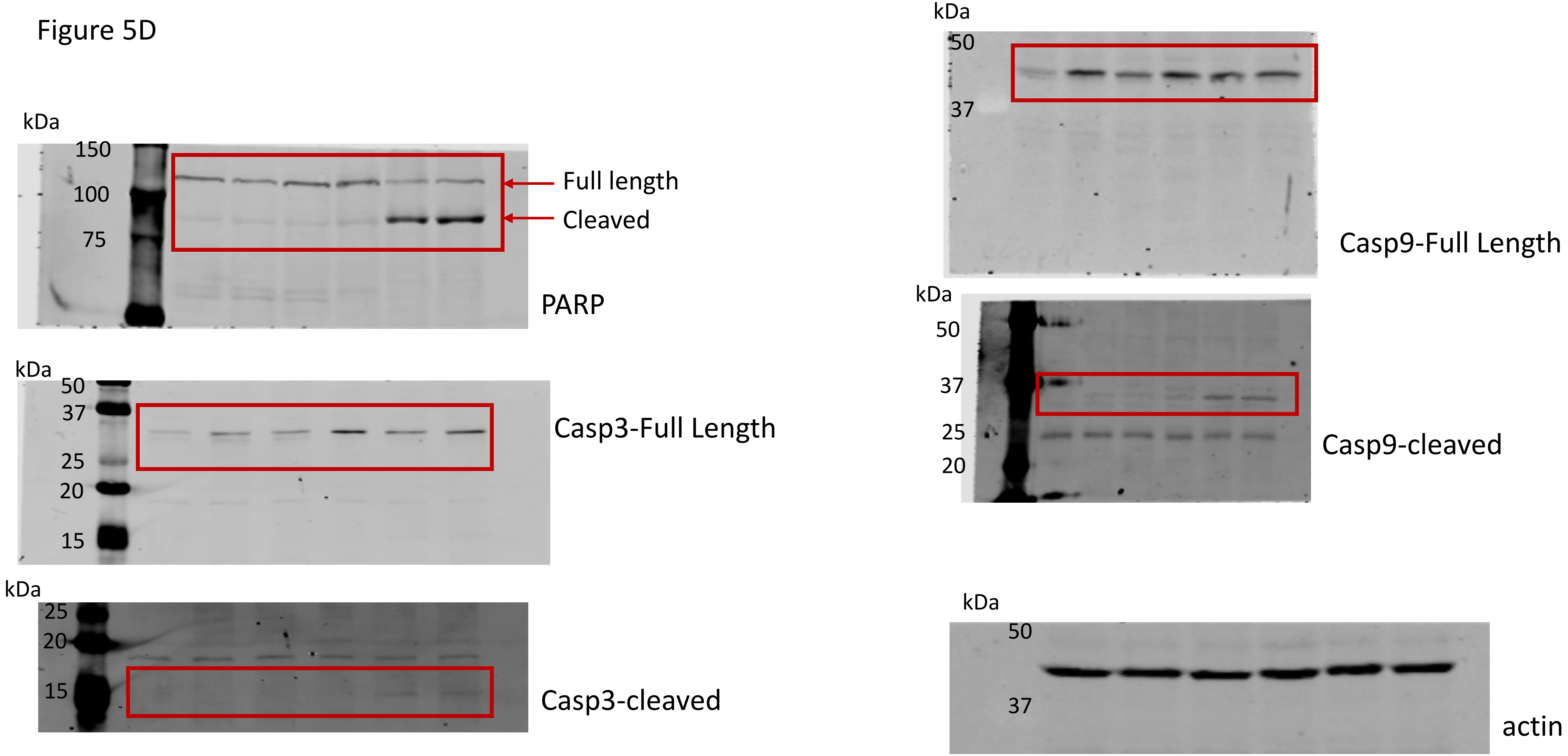
kDa
150
100
75
Full length
Cleaved
PARP
kDa
50
37
25
20
15
Casp3-Full Length
kDa
25
20
15
Casp3-cleaved
kDa
50
37
Casp9-Full Length
kDa
50
37
25
20
Casp9-cleaved
kDa
50
37
actin

Figure 6A

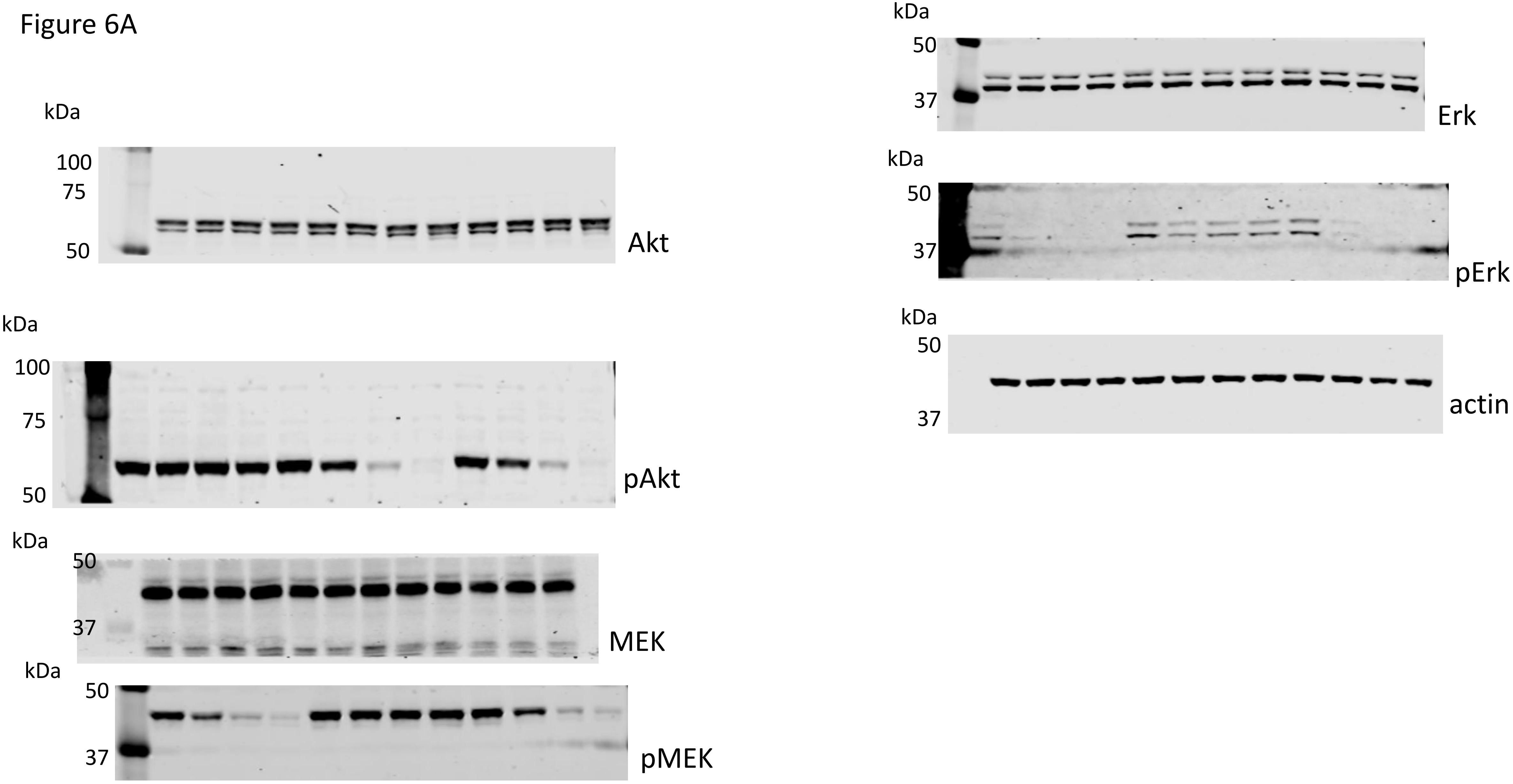
kDa
100
75
50
Akt
kDa
100
75
50
pAkt
kDa
50
37
MEK
kDa
50
37
pMEK
kDa
50
37
Erk
kDa
50
37
pErk
kDa
50
37
actin

Figure 6E

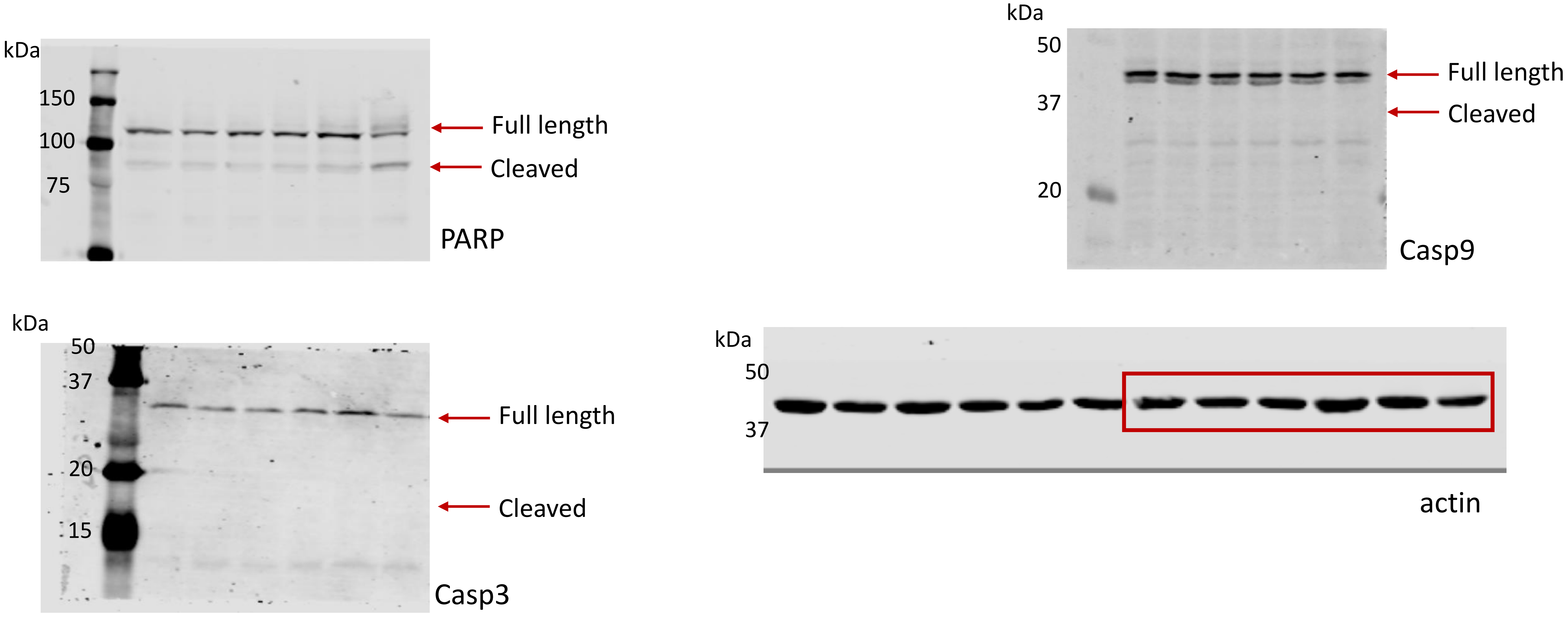
kDa
150
100
75
Full length
Cleaved
PARP
kDa
50
37
20
Full length
Cleaved
Casp9
kDa
50
37
20
15
Full length
Cleaved
Casp3
kDa
50
37
actin

Figure 7B

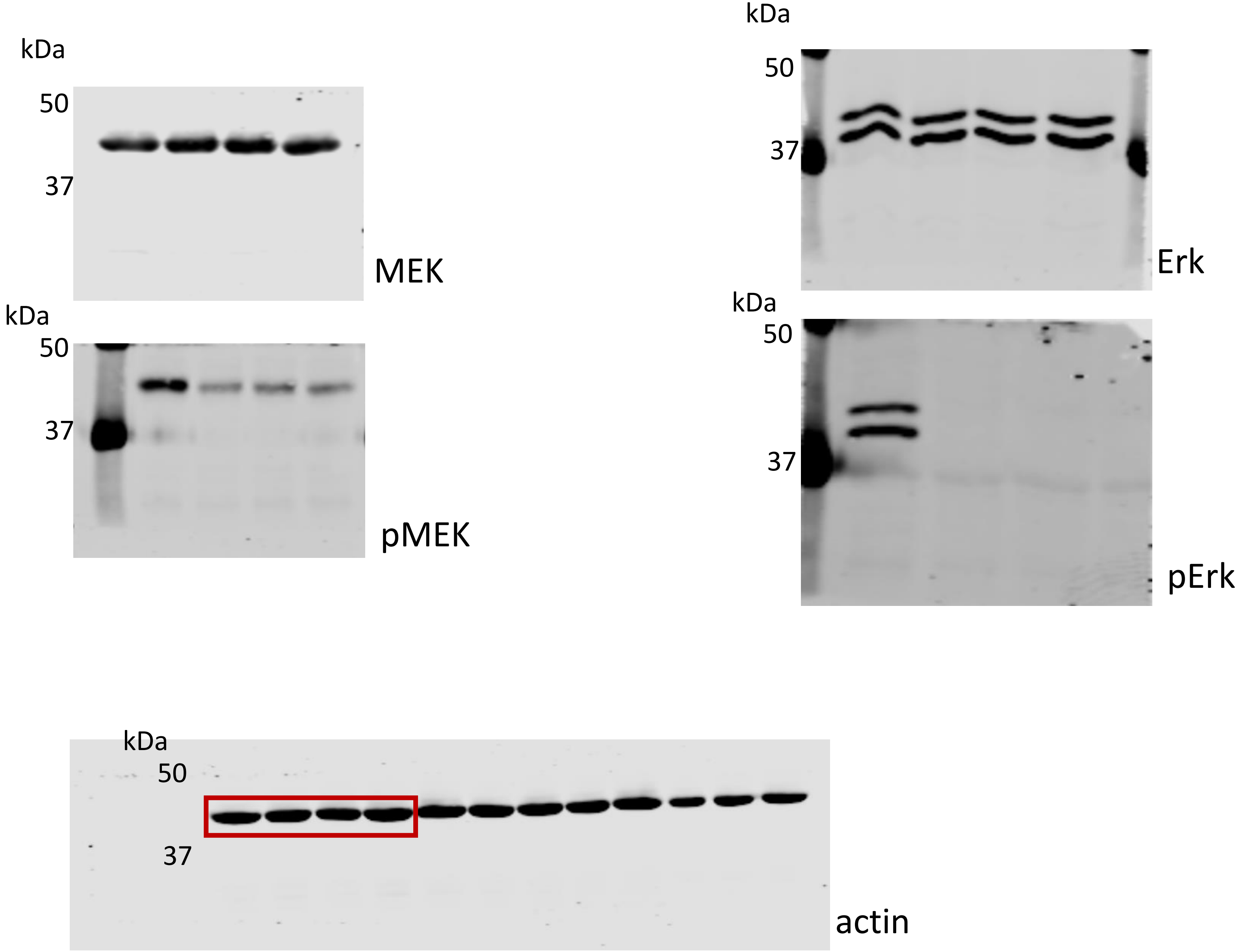
kDa
50
37
MEK
kDa
50
37
pMEK
kDa
50
37
Erk
kDa
50
37
pErk
kDa
50
37
actin
